# Realtors’ Perceptions of Social and Physical Neighborhood Characteristics Associated with Active Living: A Canadian Perspective

**DOI:** 10.3390/ijerph17239150

**Published:** 2020-12-07

**Authors:** Gavin R. McCormack, Autumn Nesdoly, Dalia Ghoneim, Tara-Leigh McHugh

**Affiliations:** 1 Department of Community Health Sciences, Cumming School of Medicine, University of Calgary, Calgary, AB T2N 1N4, Canada; dghoneim@ucalgary.ca; 2 Faculty of Kinesiology, University of Calgary, Calgary, AB T2N 1N4, Canada; 3 School of Planning, Architecture, and Landscape, University of Calgary, Calgary, AB T2N 1N4, Canada; 4 Faculty of Sport Sciences, Waseda University, Tokorozawa 359-1192, Saitama, Japan; 5 Faculty of Kinesiology, Sport, and Recreation, University of Alberta, Edmonton, AB T6G 2R3, Canada; abnesdol@ualberta.ca (A.N.); tmchugh@ualberta.ca (T.-L.M.)

**Keywords:** walkability, neighborhood, real estate, vibrancy, health, qualitative study, livability, physical activity

## Abstract

Realtors match home-seekers with neighborhoods that have built and social characteristics they desire to pursue active lifestyles. Studies have yet to explore realtors’ perspectives on neighborhood design that supports active living. Using qualitative description, our study was to explore the perceptions and understandings of neighborhood design (walkability, healthy, bike-ability, vibrancy, and livability) among urban residential realtors. Nineteen (6 men; 13 women; average age 48 years) self-identified residential realtors from Calgary, Edmonton, and Lethbridge (Canada) completed semi-structured telephone interviews. Content analysis identified themes from the interview data. Specifically, walkability was described as: perceived preferences, destinations and amenities, and connections; a healthy community was described as: encourages outdoor activities, and promotes social homogeneity; bike-ability was described as: bike-ability attributes, and was controversial; vibrancy was described as: community feel, and evidence of life; and livability was described as: subjective, and preferences and necessities. Our findings can inform the refinement of universal definitions and concepts used to in neighborhood urban design.

## 1. Introduction

There is growing interest in the role that neighborhood environments have in supporting active living and health [1,2]. The evidence suggests that neighborhood built characteristics such as higher residential and population densities, more land use and destination mix, and higher street network connectivity are supportive of physical activity, in particular walking and cycling [3,4]. Regular physical activity is associated with a reduced risk of cardiovascular disease, type 2 diabetes, hypertension, cancer, depression, and overweight and obesity [5,6]. Therefore, designing neighborhoods with built environments that support and encourage physical activity has the potential to improve population health [7,8].

Home-seekers (buyers and renters) demand certain neighborhood characteristics, and land developers supply neighborhoods with particular built characteristics, however it is realtors who are vital in matching home-seekers with their desired neighborhoods. In the U.S., 89% of buyers purchase their home through a realtor [9]. The responsibility of matching home-seekers with their desired neighborhoods requires that realtors have an understanding of the neighborhood characteristics that support and or hinder particular lifestyle preferences (e.g., physical activity). This is particularly important given that in some studies almost one-quarter of adults report that they would prefer to reside in a neighborhood with a more walkable environment [10,11]. Thus, it is important for realtors to be familiar with the concepts and terminology used in academia, advertising, media and by home-seekers themselves to describe different neighborhood built environments (e.g., walkability, bike-ability, vibrancy, livability, and healthy).

The underlying reasons for relocating to a new neighborhood are complex and numerous, but often relate to housing characteristics such as affordability and size [12]. Nevertheless, other important factors can inform residential relocation decision-making such as life-events (e.g., change in marital status) and level of neighborhood satisfaction [12,13]. Neighborhood resources related to physical activity (e.g., access to physical activity opportunities, and access to stores) are also important in people’s neighborhood decision-making [14,15]. Adults with a preference for maintaining or initiating a physically active lifestyle may choose to reside in neighborhoods that allow their preferences to be fulfilled [3,15,16]. Importantly, several studies have shown that the mismatch between a current neighborhood and neighborhood preferences can negatively affect physical activity [10,17]. For example, Frank et al. [10] found higher levels of walking for non-discretionary, discretionary or any purpose to be higher among those who preferred and also resided in a highly walkable neighborhood compared to those who would prefer to live in a walkable neighborhood but resided in a low walkable neighborhood. Misalignment between home-seeker expectations and the actual neighborhood opportunities and infrastructure can have negative implications on lifestyle, including physical activity [11,17]. Thus, realtors are vital in the delivery of accurate information regarding neighborhood characteristics to home-seekers [18,19]. Despite their importance in linking home-seekers with neighborhoods, and their albeit indirect role in promoting active living, few public health related studies engage realtors [18,19,20,21].

There is a lack of consensus in the terminology and definitions used among stakeholders when describing neighborhood characteristics related to health and active living [22,23]. Thus, the aim of this study was to explore the perceptions and understandings of neighborhood characteristics associated with active living among urban residential realtors. In our study, we focused on the commonly used concepts of walkability, healthy, bike-ability, vibrancy, and livability that are used in the public health, urban design, transportation planning, and geography literature [22,23,24,25,26,27,28,29,30].

## 2. Materials and Methods

### 2.1. Study Design

Qualitative description, as described by Sandelowski [31], was the strategy of inquiry utilized within this study. Qualitative description is typically used by researchers when there is little known about the phenomenon of interest and, therefore, was an ideal strategy of inquiry for this study. This strategy of inquiry does not require researchers to adhere to a specific process of data generation or analysis [31]. However, data generation within qualitative description typically takes the form of in-depth interviews, given that researchers are striving to develop a comprehensive summary of participants’ experiences. Additionally, the dynamic process of content analysis supports researchers in summarizing the rich experiences shared by participants. Qualitative description has been identified as an ideal approach and has been successfully applied in various other physical activity-focused studies that sought to develop a detailed description of participants’ experiences [32,33]. The University of Calgary Conjoint Health Research Ethics Board approved this study (REB19-1069).

### 2.2. Recruitment

In November 2019, study recruitment materials including newsletters, blogs, and emails were distributed to professional members (including realtors) of the Realtors Association of Edmonton, the Calgary Real Estate Board, and the Lethbridge and District Association of Realtors. According to the Alberta Real Estate Board (https://www.albertarealtor.ca/), there are over 10,500 licensed realtors in the province of Alberta. Recruitment materials were also distributed to realtors via several Real Estate Agencies located within the three cities. Interested participants contacted the research coordinator who screened them for eligibility (i.e., working as a licensed real estate agent specializing in residential homes) and scheduled eligible participants for a telephone interview. Using snowball sampling [34], participants who completed interviews were asked to recommend others suitable to participate in the study.

### 2.3. Participants

Nineteen adults (6 men; 13 women) between the ages of 24 and 70 years (average age 48 years) participated in this study. All participants self-identified as residential realtors and lived in one of three western Canadian cities (Calgary: 8 participants, Edmonton: 7 participants, and Lethbridge: 4 participants). Participants each had their Realtor license and had been practicing in the real estate industry for between 7.5 months and 40 years (average of 15 years). The majority of participants had some form of post-secondary training. Given the scope of the study, the specificity of our interview questions, data collection via in-depth, semi-structured, one-to-one interviews, and our focus on a specific target population, we set an initial minimum sample size target of 10–15 participants. Our sample size of 19 was sufficient to reach information redundancy and is similar to sample sizes in other health-related interview-based research [35].

Researchers using a qualitative description approach typically sample participants based on factors that support the researchers in addressing their study aim [31]. In the case of this study, it was essential that participants had expertise in residential real estate. In addition to specializing in residential real estate, three of the participants specialized in multiple designations, including agriculture and commercial real estate. To protect the anonymity of participants, pseudonyms have been used in the reporting of results. To this end, the participants are referred to as: Michelle, Beviel, Angela, Franny, Debbie, Kristen, Gina, LeAnne, Keith, Shannon, Rob, Lana, Wayne, Tabitha, Mary, Tamera, Chris, Tamara, and Devon.

### 2.4. Data Generation

Data were generated via one-on-one semi-structured interviews. Interviews are used in qualitative description studies to understand the nature and shape of participants’ experiences [31]. The semi-structured interview guide was informed by previous studies that have investigated the use and understanding of concepts related to the built environment and health (e.g., “walkability”) among various stakeholders [36,37]. The interview guide consisted of 6 main questions each focused on the key neighborhood related concepts of walkability, healthy, bike-ability, vibrancy, and livability (see Supplementary Materials). Given the semi-structured nature of this interview guide, the interviewer had the flexibility to probe participants to elaborate on specific details and contexts provided in their responses to the questions from the guide.

All interviews were conducted via telephone. Telephone interviews were convenient for participants and facilitated our team’s success at recruiting participants who were geographically dispersed across the province. Telephone interviews provide ideal opportunities for participants to deeply engage in research [38], and accumulating evidence suggests that telephone interviews and face-to-face interviews generate similar results [39]. A research assistant (A.N.) with expertise in qualitative research approaches and research interests focused on the psychosocial aspects of physical activity participation, conducted the interviews. Interviews lasted an average of 45 min and took place between November 2019 and February 2020. All interviews were audio-recorded and transcribed verbatim.

### 2.5. Data Analysis

Content analysis has been identified as an ideal process of analysis for qualitative description studies [31]. Researchers who employ a qualitative description approach are committed to staying “close to the data” and presenting a comprehensive summary of findings. Content analysis involves a process of distilling words into categories or themes of information, which provide a summary of phenomena [40]. As such, we employed a three-step inductive content analysis process which included: preparation, organizing, and reporting [40].

Criteria used to evaluate qualitative research are dynamic, and depend on the specific research purpose and context [41]. Within this qualitative description study, multiple strategies were used to enhance the trustworthiness of findings. Methodological coherence, as described by Morse et al. [42], was achieved by ensuring that there was alignment among the study design (i.e., qualitative description), research questions, sampling, data generation, and analysis. Additionally, consistent with a qualitative description study design, the inclusion of rich, thick descriptions of participants’ experiences were provided in the reporting of findings. Creswell [43] explained how rich, thick descriptions are one of the primary processes of verification in qualitative study designs. Finally, the inclusion of multiple researchers in the data analysis contributed to a process of triangulation, which Creswell also described as a key method of verification in qualitative studies.

## 3. Results

The experiences shared by realtors provide in-depth insight into the ways in which the concepts of walkability, healthy, bike-ability, vibrancy, and livability are understood within the context of neighborhoods. The realtors described these concepts from their own perspective and experiences, the perspectives from the real estate profession in general, and from the perspectives of the home-seekers with whom they engage. The various terms are represented by themes, which are supported by direct quotes from participants. Specifically, walkability was described as: (a) perceived preferences, (b) destinations and amenities, and (c) connections; a healthy community was described as one which: (a) encourages outdoor activities, and (b) promotes social homogeneity; bike-ability was described as: (a) bike-ability attributes, and (b) controversial; vibrancy was described as: (a) community feel, and (b) evidence of life; and livability was described as (a) subjective, and (b) preferences and necessities. It is important to acknowledge that there is overlap among the themes, and the themes are not mutually exclusive.

### 3.1. Walkability

Realtors indicated that they rarely used the term walkability. However, many have seen the term online, in the form of Walk Score^®^. The realtors that had heard and used the term walkability among their home-seekers, suggested that the term is most popular among young first-time and senior home-seekers. Walkability themes that emerged included: (a) perceived preferences, (b) destinations and amenities, and (c) connections.

#### 3.1.1. Perceived Preferences

The term walkability was described by participants as a subjective term and one that depends on perceived preferences of home-seekers. As stated by Michelle, “I think it is a personal definition. My ten blocks of walkability is not the same as others… others may not think that is a walkable location”. Similarly, Beviel said, “A realtor has to ask questions about the lifestyle of a client, or how they want to be… So, it depends on the priorities of the people as to what I would recommend… It depends on the lifestyle”. Beviel further explained that different people have different understandings and preferences regarding walkability, and it is necessary for realtors to understand home-seeker preferences: “there are some places that have a type of walkability where people can experience outdoors and are close to facilities that they might use. Other areas are quite far out where you would have the experiential type [of walkability], but you couldn’t walk to a store. So… it just depends what people mean by walkability, I guess”.

Given the subjectivity of the term, Angela described the importance of asking home-seekers questions about walkability. She said, “If I am talking to clients I will get into what walkability means to them… Do you like to go for a walk, or walk to amenities in any season?” Recognizing that walkability means different things to different people, Franny argued that there is a need for consistent messaging around the term. She said, “I have seen it [the term walkability] in a lot of contexts, and it is not necessarily very consistent for the consumer. If the term was used with a very clear point A to point B, I think the message would be clearer to consumers”.

#### 3.1.2. Access to Services and Amenities

Realtors described how access to various services and amenities contributes to neighborhood walkability. For instance, access to stores, restaurants, transit, and parks or greenspace were described as key features of walkability. Participants also described how safety and aesthetics are important to consider within the context of access to services and amenities. Angela’s description of walkability serves a detailed example of the importance of access to services and amenities: “I would say the ability to walk to several different amenities within a community. So not just walking paths, but being able to walk to a grocery store, not just a corner store, but drug stores, restaurant, pubs, schools, transit. In addition to having some nice streetscapes, parks, or areas like that to walk around… I think with walkability there are a lot of factors, but I think it does have to have the 5 main things: grocery stores, transit, restaurants, shops, and parks that kind of public spaces. I think it is really important”. Similarly, Debbie echoed this sentiment in her description of walkability: “A walkable neighborhood is just minutes to where you are going to take your children to school, to dentist, to doctors, to community activities. If you are in the inner city and you do not have access to a vehicle it is really important for work”.

While some realtors described walkability in detail, others were succinct. For instance, Kristen described walkability as “the access that someone has to services within a neighborhood by simply walking”. Similarly, Gina explained how walkability means that people have access to amenities, pathways, and transit opportunities. Gina also suggested that the term “accessibility” might be a more useful term because “walkability comes with the assumption that you can walk”.

Within the context of access to services and amenities, LeAnne also explained how safety is a key feature of neighborhood walkability. She explained: “Walkability in my mind would be a location that is safe, developed, or close to green spaces. Generally, something that is inviting for your health and your sense of community. I would expect it to be something with trees, and shops, and a welcoming feel”. Safety of pathways was also mentioned by various realtors. As LeAnne explained, “when I mention walkability to my clients, there is an element of safety”. Safety was discussed in terms of well-lit pathways and neighborhoods at night, cleared pathways in the wintertime, and smooth and continuous routes.

#### 3.1.3. Connections

The realtors suggested that connection, including both physical and social connections, are an important feature of walkability. In terms of physical connections, Keith explained that a walkable neighborhood means that “there are great pathways, shared use pathways, very clear and numerous pathways for pedestrians and cyclists connecting streets through green strips and parks leading you out to different service areas and different facilities”. Additionally, Shannon and Michelle suggested that such physical connections support necessary transit options. Shannon indicated that for neighborhoods to be walkable, they need to facilitate the ability to get to a destination “via public transit, as it could take care of any other leg of the journey you might need” when not on foot. Similarly, Michelle explained that “transit would go hand in hand with walkability”.

In addition to the physical connections, social connections were also described as a key feature of a walkable neighborhood. For instance, walkable neighborhoods encouraged a sense of community. As Rob suggested, “the more that you get out and about the more that you’ll chat with neighbors and foster a sense of community in the neighborhood”. Similarly, LeAnne explained how a neighborhood that has low walkability would be “empty of personality” because the neighborhood would be void of people walking around and there would be “no participation in the neighborhood”.

### 3.2. Healthy

The term healthy, within the context of neighborhoods, was difficult for the realtors to describe. They suggested that the term healthy was not typically used when referring to neighborhoods, but used more often in describing a house or property. As Lana stated, “I don’t think they [real estate professionals] would use the word healthy, but they might advertise a lifestyle”. Despite the challenges in describing the term, participants’ shared experiences suggest that a healthy neighborhood: (a) encourages outdoor activities, and (b) promotes neighborhood social homogeneity.

#### 3.2.1. Encourages Outdoor Activities

When referring to a healthy neighborhood, the realtors emphasized the health benefits of being able to walk or bike to local services and amenities. They argued that healthy neighborhoods have accessible amenities and aesthetic and appealing features. Beviel said, “I would think one that has a lot of vegetation. Trees and plants. Because that helps the environment, but it also helps people both aesthetically and with how they perceive it affects their lifestyle”. Several realtors discussed the condition of neighborhood parking as a deterrent of health. As Shannon suggested, “healthy to me would be making sure that we don’t have over-parked streets”. She argued that having too much traffic in a “tighter neighborhood” is not healthy.

When asked about what constitutes a healthy neighborhood, Rob stated, “the first thing that comes to mind for me, does the neighborhood design encourage people to be outside on a regular basis? Going for walks, biking, being outdoors”. Similarly, Wayne explained: “A healthy community is the pathways are accessible. Walking to a neighborhood pub, park, coffee shop… it’s accessible. The areas that people want to go to are accessible by bicycle. A neighborhood that encourages people to get out and move, by bike or walking, to amenities in their community rather than driving. For a community to be healthy it needs to be accessible and planned properly… older areas are limited to what they can do to get that to a higher level of healthy”. Keith shared similar sentiments to Wayne, but Keith also highlighted the importance of safety. He said, “You have to feel safe, be inviting. It’s going to attract you and motivate you to get out and move and go for a walk. I guess a part of health is feeling safe as well”.

#### 3.2.2. Promotes Social Homogeneity

Realtors provided numerous examples to suggest that neighborhood social homogeneity is a key feature of a healthy neighborhood. As Beviel suggested, “I think a healthy neighborhood would have conveniences that are fairly close, and similar houses… You don’t want to have a $200,000 home next to a $600,000 house. [Our city] has tried that, and it just doesn’t work. So, if you have a grouping of say $250,000 houses, most of the people who are buying in that area are in a like frame of lifestyle. They might have families; they might have pets. Whereas people, perhaps my age… I don’t want to be living by a bunch of screaming kids (laughs)”. Tabitha argued that she does indeed appreciate when houses are not all of the same design within a community but, similar to Beviel, she said, “I don’t think you should have a house worth $300,000 and a house worth $800,000 on the same street. Maybe a $100,000 to $200,000 spread but not an $800,000 spread”.

When describing healthy neighborhoods, Keith explained how he tries to describe the overall “condition of the neighborhood”. He explained, “Nobody really says, ‘I want to live in a healthy neighborhood’. But if we are talking about the attributes, we might talk about neighborhoods that are, or homes that are, clean and well kept. There is a sense of pride of ownership in neighborhoods, uncluttered. That all speaks to a sense of healthiness. So, I guess on that front we, in a roundabout way, talk about healthy neighborhoods with just about every buyer or client that we sit down with”. Mary further elaborated by suggesting that the condition or health of a neighborhood can be compromised when there are rental properties or multi-family homes. In describing what constitutes a healthy neighborhood she said, “When properties all conform to the same ideals and you know that even if there is a vacant lot beside you, which sometimes there is, the likelihood of a duplex or a multifamily being built on that lot next to you is slim to none. Because that is important as well, because people that spend good money for a nice home do not want a rental property next to them”.

### 3.3. Bike-Ability

The realtors described how bike-ability is very similar to walkability. When describing bike-ability Keith asked if it was “similar to walkability but are there cycling or multi-use paths that help a cyclist move with, or through, traffic with a sense of purpose or direction?” Specifically, bike-ability was described by participants as: (a) bike-ability attributes, and (b) controversial.

#### 3.3.1. Bike-Ability Attributes

Bike paths and multi-use trails were key features that the realtors described as necessary for a neighborhood to be bike-able. For example, Beviel suggested: “I think it has to do with designated pathways for bikers, and places they can be parked if you are going somewhere on a bike. For instance, bike stands in locations. But mostly I believe it is having the road work and the plan, so bikes can maneuver around the city”. Similarly, Rob explained how a bike-able neighborhood would have a “paved trail system nearby, with access to the neighborhood. Close enough proximity so you can bike to amenities maybe connecting other neighborhoods as well”. In describing how she refers to bike-ability with home-seekers, Debbie explained how “we talk about it for recreational purposes, for exercise. They want to be able to go out for a ride”. Realtors also acknowledged that some people want a bike-able neighborhood for more than just exercise. As stated by Angela, “I have a lot of friends that are into cycling and they love the coffee shops that are popping up for people on bikes to stop and hang out and have a coffee”.

#### 3.3.2. Controversial

Realtors described how promoting a neighborhood as bike-able is sometimes controversial. For instance, participants voiced how some home-seekers are frustrated about the infrastructure that supports biking. For example, Tamera shared: “Our bike lanes here are fairly new and extremely controversial… there is less parking now and maybe a few more minutes added to a commute because of light and traffic changes. We have lost two traffic lanes and several parking spots. There is also that you have to look for bikers when turning right. So, it adds a few minutes and is a little bit annoying. But in my opinion, it is for the better. But it is something that is discussed often”.

Taking a similar stance, Chris said “I’m not a big fan of losing a lane for a bike lane, but I think it is important to feel safe so you can cycle”.

### 3.4. Vibrancy

Overall, a vibrant neighborhood was described using adjectives such as active, interesting, and new. Young first-time and senior home-seekers were said to be the most interested in living in vibrant communities. Although participants acknowledged that the concept of vibrancy might be somewhat subjective, their shared experiences suggest that vibrancy includes: (a) community feel, and (b) evidence of life.

#### 3.4.1. Community Feel

In terms of the feel of a community, Rob explained, “I guess with vibrant, they [houses] are generally quite new. I look at my neighborhood and some of the houses are getting old so that would be the number one reason I would describe it as not vibrant, just because it is not up and coming”. Similarly, Tamara described neighborhood vibrancy as “the look, the flow, and the feel of a community. It is brand new, with that new modern style; the look of the building catches your eye. It pops”.

Lana shared Tamara’s sentiments that a vibrancy suggests a new community but also noted the preference of vibrant communities among younger adults: “They will say it is a vibrant new neighborhood… it’s typically marketed towards younger families as being something that is exciting, something that young people can get their teeth into… y’ know, even condominiums will sometimes use that in new neighborhoods… Saying that it’s a really vibrant community, it has a lot of amenities and things for them to do”. Similarly, Devon said that a “higher vibrancy might mean more younger people, more educated professionals. I also think about is it close to bars and nightlife? Those are the two things that come to mind in terms of vibrancy”.

Although some of the participants explained that vibrant neighborhoods typically consist of “younger” people, Tamera argued that this is not always the case. In working with seniors, Tamera explained, “Especially those who don’t feel like they are ready to settle down yet but are looking to downsize… They don’t want to be in an old folk’s community because they want more young things to do. Even if there are seniors’ leagues, like bridge I don’t think that would be vibrancy per se… vibrant would be more like clubs, community, or social events”. While not referring specifically to a target population, Shannon did acknowledge that a vibrant neighborhood is one in which there is “lots to do”. She said, “vibrancy is being able to pop out of your home and be able to get to a nice little restaurant”.

#### 3.4.2. Evidence of Life

Participants described vibrant neighborhoods as those that have numerous amenities and services, and those that have “evidence of life”. They explained how services, amenities, and design features comprise a vibrant community. As stated by Franny, it is the “proximity to different types of amenities that kind of brings the community to life”. Franny further explained that the concept of vibrancy is quite ambiguous, however she provided examples of what constitutes a vibrant community. She said, “Although the word vibrancy specifically, would lend more to the faster paced side but I guess it would depend on the context of the person writing the ad. This one [the term vibrancy] has more ambiguity; it is more arbitrary. It is too subjective. More of an adjective than a term to rate the community. It is an advertisement word. It is the tourism of the community. That is what I would define it as. The opportunity for livelihood. Amenities that foster livelihood in the community”. Beviel shared similar sentiments to Franny. However, while Beviel did not highlight the ambiguous nature of vibrancy, she explained how vibrancy is related to the “life” of a community: “Many older areas just have rows and rows of streets. There is no life to it, there is no reason for people to be out of their homes and associating outside. So, it just seems it is just houses. Whereas if you have a community that is designed where there are parks, places for people to go and perhaps picnic outside. There is more life that seems to be going on, and people are spending time in a community”.

While some participants were focused on the specific amenities and services that contribute to vibrancy, others focused more on the design features of a community. As Keith explained, “To me when you say that I think of neighborhoods that have visually attractive features. I think architecture… When I think of vibrancy, I think of going through a neighborhood and seeing people enjoying the neighborhood and features—out there doing activities. Evidence of life”. While Keith’s initial description of vibrancy was focused on the attractiveness of a neighborhood, he also explained how vibrant communities have “lots of community events, lots of events that engage the community. Vibrancy… that’s the first thing that comes to mind. Different events, community events, festivals. That kind of thing”. Expanding on this, LeAnne suggested that “what creates vibrancy is people’s awareness of what it is going on in a community. Y’know people are aware of events that they can go to and participate in. It is what brings people out”. Similarly, Franny suggested that a vibrant neighborhood “promotes interaction between residents like a lake or a restaurant. That the community only consists of residents would be non-vibrant not close to anything”.

### 3.5. Livability

Realtors suggested that the concept of livability is an all-encompassing term, which includes walkability, bike-ability, vibrancy, and healthy. When asked about livability, many participants did not have much to say because they felt that this concept was covered by discussion of the other concepts, although some realtors did suggest that that concept of livability was linked with the age or life-stage of the home-seeker. Beviel states, “I would think that it incorporates all the things that we have talked about. If it’s a livable neighborhood you have all of those amenities you have a combination of everything it makes it more enjoyable to live there”. Wayne further expanded by saying: “I think that the problem here is that we have already covered this”. Despite the participants’ acknowledgement that livability was discussed within the context of the other concepts, participants described livability as: (a) subjective, and (b) preferences and necessities.

#### 3.5.1. Subjective

Livability is a concept that realtors described as subjective, and that typically depends on a person’s stage of life. As Keith stated, “Livable is relative to where you are in your life. I am speaking from a midlife more established perspective and less congestion is more livable…the definition changes as you go through the life cycles”. Michelle shared similar sentiments: “All of my clients would define this [livability] differently. Livability for some young people would be close to a bar, livable for some older people would be close to an amenity where they can make new friends like a movie theatre or library. Like I said, in my late 30s I want green space or park space for my children. I don’t think that I have one way of defining it that is right or overarching”. Recognizing that livability is a concept rather than just a word, Beviel said, “I think it is more of a concept rather than what some people… y’know when they walk into a neighborhood they say ‘this is nice, I like it here’ but they don’t say the term livable. So, it’s more of a concept in your head. Again, a lot of it depends on age, lifestyle, things that people do for hobbies… all of that is wrapped up into it, whether they think a neighborhood is livable or not”.

#### 3.5.2. Preferences and Necessities

When describing the term livability, realtors provided numerous examples to suggest that the concept comprises preferences and necessities. Notably, some realtors viewed livability as what some scholars might describe as the “habitability” [44] of the neighborhood and buildings and the provision of basic services. As Tabitha stated, “Oh gosh that [livability] could be so many things. A neighborhood you like and feel safe in, somewhere you want to be. But then, on the other hand, it could be it has running water, and heat, its outdated… but it’s livable”. In terms of preferences, Tabitha explained that a livable community is a place where someone “wants to be” and, at the same time, a livable community has certain necessities in terms of running water and heat. Michelle built on this notion by saying that a home or community is considered livable when it has the necessities: “We usually use the term livable when the community is older, and the home is older. It could have been updated, but you are usually just buying the home for land value. The home is still livable with or without upgrades, the price is for land value”. To further elaborate on the idea that livability reflects preferences and necessities, Shannon stated, “For most people it has got to have access to groceries, healthcare, medical, recreation area… all of those things make a neighborhood way more livable”. Echoing this sentiment, Rob suggested: “The location, proximity to surrounding amenities is important for livability. Not necessarily like walkability, where you can walk there but I’d say the fact that you are not isolated. Like you don’t have to travel a great distance to the places that you want to be on a regular basis. That is kind of what comes to mind for me”. Similarly, Lana explained, “It [livability] means that there will be a grocery store nearby, there will be a dentist near by… a vet… and all that stuff you would need. So, that again, you can stay within your community and not have to leave that area in order to get everything that you may need”.

## 4. Discussion

Our study explored realtor perspectives on concepts commonly used in public health, urban design, transportation planning, and geography to describe neighborhood design associated with active living. Among realtors, multiple themes emerged for the neighborhood design concepts of walkability (perceived preferences; destinations and amenities; and connections), healthy (encourages outdoor activities; and social homogeneity), bike-ability (bike-ability attributes; and controversial), vibrancy (community feel; and evidence of life), and livability (subjective; and preferences and necessities). Our findings, which are grounded in the experiences of the participants, highlight important considerations for developing consistent definitions and awareness of concepts among realtors to ensure home-seekers are matched with neighborhoods that support their preference for an active lifestyle.

Among realtors there was general agreement in the meaning and understanding of concepts related to the neighborhood environment. Despite realtors reporting they did not often use terms such as walkability, bike-ability, healthy, vibrancy, and livability when communicating with home-seekers, they did identify specific neighborhood built and social characteristics that support physical activity, sense of community, and health of residents. For instance, the realtors identified proximity to services and amenities, availability of greenspace and parks, availability of transit, and safety (lighting and cleared paths) as important built characteristics contributing to neighborhood walkability. These built characteristics were similar to those identified by Edmonton residents as contributing to walkability (e.g., proximity to amenities and services, safety, available paths, greenspaces) [45]. Realtors also identified proximity to services and amenities, cycling infrastructure (e.g., paths), bicycle parking facilities, avoidance of traffic, and connectivity including routes facilitating inter-neighborhood travel as important built characteristics contributing to the bike-ability of a neighborhood. In support of previous findings [46,47], walkability was considered to be important for facilitating a sense of community and social capital via social interactions among residents.

Realtor perspectives on the built characteristics that contribute to walkability and bike-ability align with those characteristics found to be supportive of walking and cycling in previous studies [3,4,48,49,50]. However, although these built characteristics are important to support walking and biking, according to U.S. realtors, affordability and value, safety from crime, and the quality of schools are the most important considerations by home-seekers, suggesting that walkability and bike-ability might be less influential on neighborhood choice [21]. Notably, realtors identified bike-ability as controversial. Other studies have noted that non-cyclists often have negative attitudes towards the investment in and development of cycling infrastructure, as well as towards cyclists themselves [51,52]. Diffusion of evidence-informed knowledge regarding bike-ability from realtors to home-seekers could increase awareness about the importance of bike-ability and legitimacy of cycling as a transportation mode among the public. Notably, personal biases of realtors about specific neighborhood characteristics (e.g., negative perceptions towards bike-lanes) could influence neighborhood choice of home-seekers even if home-seekers had not previously considered these characteristics as important in their decision-making. This highlights the need for objective and accurate neighborhood data that are easily accessible to realtors (e.g., walkability index, bike-ability index, crime index, and park or greenspace index) and for realtors to use these data, instead of personal opinions, to educate and inform their clients. Publicly available metrics reflecting neighborhood supportiveness for walking, transit use, and cycling (Walk Score^®^, Transit Score^®^, and Bike Score^®^, respectively) are available to North American realtors via real estate websites (e.g., MLS^®^; REDFIN). The extent to which realtors use these metrics when communicating with home-seekers remains unknown.

Relative to the other concepts, healthy, vibrancy, and livability took on more abstract meanings for realtors. Realtors noted that the concepts of healthy and livability reflect a neighborhood’s propensity to allow residents to achieve a self-determined lifestyle. Healthy neighborhoods were considered to be those with built characteristics that encouraged walking and cycling and other outdoor activity, and those with trees and vegetation, low traffic, and maintained homes. Several reviews report associations between neighborhood built characteristics and health outcomes including chronic disease and quality of life [46,47,53,54]. Realtors also perceived several social characteristics contributing to a healthy neighborhood including evidence of homeowner pride, residents with the same ideals, similar home values, and social homogeneity (i.e., residents with similar income, education, and ethnicity). This finding might suggest that realtors perceive more socially homogenous neighborhoods to be healthier. System-level factors are known to contribute to residential segregation (e.g., housing and mortgage discrimination) [55,56], however our findings suggest that the biases of some realtors could also contribute to residential segregation, especially if home-seekers express desires to live in a “healthy neighborhood”. Residential segregation can result in poorer health and exacerbate health inequalities, especially among minority and economically disadvantaged populations [55,57,58].

Like healthy and livability, realtors associated vibrancy with the social characteristics of a neighborhood. They noted that more vibrant neighborhoods seemed to have newer homes with younger and highly educated residents. Vibrant neighborhoods included those that had clubs and community events, where residents were happy and enjoying activities in and around the neighborhood. Vibrancy was related to local amenities, because they facilitated socializing. Realtors’ perspectives on vibrant neighborhoods having a community feel and demonstrating evidence of life is congruent with traditional notions of neighborhood or urban vibrancy which reflects levels of street life, intensity of activity, and concentrations of people, facilitated by higher densities and a mix of local amenities and services [59,60]. Like others [61], realtors recognized livability as an all-encompassing concept that can mean different things depending on an individual’s stage of life, but acknowledged that livability of a neighborhood is related to the provision of infrastructure and services to support daily life and active living.

Our study has several limitations. Realtors self-selected to participate in the study, and therefore our findings may reflect the perspectives of those with the strongest opinions on the study topic. Our sample included urban realtors from three major cities in a western Canadian province, and therefore the perspectives of realtors in our study may not be transferrable to rural areas or other countries. For instance, walkability, bike-ability, healthy, vibrancy, and livability may have different meanings for realtors (and home-seekers) in different cities because of differences in urban designs, norms (i.e., cultural, social, and behavioral), and health behaviors (e.g., walking, cycling, and transit use). Moreover, the terminology used in our study may not be universal to all populations or contexts. Our interview questions focused on walkability, healthy, bike-ability, vibrancy, and livability, however other concepts and terminology exist for describing neighborhood design in relation to active living (e.g., new urbanist, traditional, conventional, age-friendly, pedestrian-friendly, sustainability) that might be used more frequently used among realtors and their clients. Due to our geographically dispersed population and the nature of our interview questions, we used telephone-administered semi-structured interviews. Although outside the scope of our study, focus groups might be a useful qualitative approach for identifying and developing common terminology and concepts related to neighborhoods among groups of realtors.

## 5. Conclusions

Our findings suggest that realtors consider built and social environmental characteristics important for creating walkable, bike-able, healthy, vibrant, and livable neighborhoods, but the nature of these characteristics might differ depending on home-seeker sociodemographic characteristics and stage of life. Our findings also suggest that realtors do attempt to match home-seekers with their desired neighborhoods, including home-seekers wanting to reside in walkable, bike-able, healthy, vibrant, and livable neighborhoods, however, realtor biases regarding home-seeker sociodemographic characteristics (e.g., age, income, and ethnicity) and the belief that neighborhood social homogeneity is an indicator of a healthy neighborhood has the potential to undermine the matching of home-seekers with neighborhoods that support active living. Future studies should explore how realtor perspectives on neighborhood characteristics inform neighborhood choice decisions among home-seekers and in turn how these decisions affect home-seeker health. Future studies with home-seekers might provide interesting and complementary insights into how this group perceives walkable, bike-able, healthy, vibrant, and livable neighborhoods, perceived influence of realtors, and how these perspectives contribute to their neighborhood choice decision-making. Our findings contribute knowledge that can inform the development of universal definitions and understanding of terminology and concepts used to describe neighborhood urban design among stakeholders, including realtors and home-seekers.

## Supplementary Materials

The following are available online at https://www.mdpi.com/1660-4601/17/23/9150/s1.

## References

[1] Giles-Corti B., Sallis J.F., Sugiyama T., Frank L.D., Lowe M., Owen N. (2015). Translating active living research into policy and practice: One important pathway to chronic disease prevention. J. Public Health Policy.

[2] Koehler K., Latshaw M., Matte T., Kass D., Frumkin H., Fox M., Hobbs B.F., Wills-Karp M., Burke T. (2018). Building Healthy Community Environments: A Public Health Approach. Public Health Rep..

[3] McCormack G.R., Shiell A. (2011). In search of causality: A systematic review of the relationship between the built environment and physical activity among adults. Int. J. Behav. Nutr. Phys. Act..

[4] Ferdinand A.O., Sen B., Rahurkar S., Engler S., Menachemi N. (2012). The relationship between built environments and physical activity: A systematic review. Am. J. Public Health.

[5] Warburton D.E.R., Bredin S.S.D. (2017). Health benefits of physical activity: A systematic review of current systematic reviews. Curr. Opin Cardiol..

[6] Reiner M., Niermann C., Jekauc D., Woll A. (2013). Long-term health benefits of physical activity—A systematic review of longitudinal studies. BMC Public Health.

[7] Northridge M.E., Sclar E.D., Biswas M.P. (2003). Sorting out the connections between the built environment and health: A conceptual framework for navigating pathways and planning healthy cities. J. Urban Health.

[8] Durand C.P., Andalib M., Dunton G.F., Wolch J., Pentz M.A. (2011). A systematic review of built environment factors related to physical activity and obesity risk: Implications for smart growth urban planning. Obes. Rev..

[9] National Association of REALTORS® (2019). 2019 Profile of Home Buyers and Sellers.

[10] Frank L.D., Saelens B.E., Powell K.E., Chapman J.E. (2007). Stepping towards causation: Do built environments or neighborhood and travel preferences explain physical activity, driving, and obesity?. Soc. Sci. Med..

[11] Badland H., Oliver M., Kearns R.A., Mavoa S., Witten K., Duncan M.J., Batty G.D. (2012). Association of neighbourhood residence and preferences with the built environment, work-related travel behaviours, and health implications for employed adults: Findings from the URBAN study. Soc. Sci. Med..

[12] Clark W.A.V., Huang Y. (2003). The life course and residential mobility in British Housing markets. Environ. Plan. A.

[13] Clark W.A.V., Deurloo M.C., Dieleman F.M. (1984). Housing Consumption and Residential Mobility. Ann. Assoc. Am. Geogr..

[14] McCormack G.R. (2017). Neighbourhood built environment characteristics associated with different types of physical activity in Canadian adults. Health Promot. Chronic Dis. Prev. Can. Res. Policy Pract..

[15] Lamb K.E., Thornton L.E., King T.L., Ball K., White S.R., Bentley R., Coffee N.T., Daniel M. (2020). Methods for accounting for neighbourhood self-selection in physical activity and dietary behaviour research: A systematic review. Int. J. Behav. Nutr. Phys. Act..

[16] Cao X., Mokhtarian P.L., Handy S.L. (2009). Examining the impacts of residential self-selection on travel behaviour: A focus on empirical findings. Transporation Rev..

[17] Schwanen T., Mokhtarian P.L. (2005). What affects commute mode choice: Neighborhood physical structure or preferences toward neighborhoods?. J. Transp. Geogr..

[18] Trowbridge M.J., Pickell S.G., Pyke C.R., Jutte D.P. (2014). Building Healthy Communities: Establishing Health And Wellness Metrics For Use Within The Real Estate Industry. Health Aff..

[19] Braunstein S., Lavizzo-Mourey R. (2011). How The Health And Community Development Sectors Are Combining Forces To Improve Health And Well-Being. Health Aff..

[20] Jutte D., LeWinn K.Z., Hutson M.A., Dare R., Falk J. (2011). Bringing Researchers And Community Developers Together To Revitalize A Public Housing Project And Improve Health. Health Aff..

[21] Carnoske C., Hoehner C., Ruthmann N., Frank L., Handy S., Hill J., Ryan S., Sallis J.F., Glanz K., Brownson R. (2010). Developer and Realtor Perspectives on Factors That Influence Development, Sale, and Perceived Demand for Activity-Friendly Communities. J. Phys. Act. Health.

[22] Forsyth A. (2015). What is a walkable place? The walkability debate in urban design. Urban Des. Int..

[23] Talen E., Koschinsky J. (2013). The Walkable Neighborhood: A Literature Review. Int. J. Sustain. Land Use Urban Plan..

[24] Miller H.J., Witlox F., Tribby C.P. (2013). Developing context-sensitive livability indicators for transportation planning: A measurement framework. J. Transp. Geogr..

[25] Pandey R., Garg Y., Bharat A. (2013). Understanding qualitative conceptions of livability: An Indian perspective. Int. J. Res. Eng. Technol..

[26] Redaelli E. (2016). Creative placemaking and the NEA: Unpacking a multi-level governance. Policy Stud..

[27] Braun L.M., Malizia E. (2015). Downtown vibrancy influences public health and safety outcomes in urban counties. J. Transp. Health.

[28] Barreca A., Curto R.A., Rolando D. (2020). Urban vibrancy: An emerging factor that spatially influences the real estate market. Sustainability.

[29] Krenn P.J., Oja P., Titze S. (2015). Development of a Bikeability Index to Assess the Bicycle-Friendliness of Urban Environments. Open J. Civ. Eng..

[30] Kellstedt D., Spengler J.O., Foster M.J., Lee C., Maddock J.E. (2020). A scoping review of bikeability assessment methods. J. Community Health.

[31] Sandelowski M. (2000). Whatever happened to qualitative description?. Res. Nurs. Health.

[32] McCormack G.R., McFadden K., McHugh T.-L.F., Spence J.C., Mummery K. (2019). Barriers and facilitators impacting the experiences of adults participating in an internet-facilitated pedometer intervention. Psychol. Sport Exerc..

[33] Larson H.K., McFadden K., McHugh T.L.F., Berry T.R., Rodgers W.M. (2018). When you don’t get what you want—And it’s really hard: Exploring motivational contributions to exercise dropout. Psychol. Sport Exerc..

[34] Faugier J., Sargeant M. (1997). Sampling hard to reach populations. J. Adv. Nurs..

[35] Vasileiou K., Barnett J., Thorpe S., Young T.P. (2018). Characterising and justifying sample size sufficiency in interview-based studies: Systematic analysis of qualitative health research over a 15-year period. BMC Med. Res. Methodol..

[36] Clark M.I., Berry T.R., Spence J.C., Nykiforuk C., Carlson M., Blanchard C. (2010). Key stakeholder perspectives on the development of walkable neighbourhoods. Health Place.

[37] Stankov I., Howard N.J., Daniel M., Cargo M. (2017). Policy, Research and Residents’ Perspectives on Built Environments Implicated in Heart Disease: A Concept Mapping Approach. Int. J. Environ. Res. Public Health.

[38] Irvine A., Drew P., Sainsbury R. (2013). ‘Am I not answering your questions properly?’ Clarification, adequacy and responsiveness in semi-structured telephone and face-to-face interviews. Qual. Res..

[39] Sturges J.E., Hanrahan K.J. (2004). Comparing Telephone and Face-to-Face Qualitative Interviewing: A Research Note. Qual. Res..

[40] Elo S., Kyngäs H. (2008). The qualitative content analysis process. J. Adv. Nurs..

[41] Sparkes A., Smith B. (2013). Qualitative Research in Sport, Exercise and Health Sciences. From Process to Product.

[42] Morse J.M., Barrett M., Mayan M., Olson K., Spiers J. (2002). Verification Strategies for Establishing Reliability and Validity in Qualitative Research. Int. J. Qual. Methods.

[43] Creswell J. (2013). Qualitative Inquiry and Research Design: Choosing among Five Approaches.

[44] Jacobs D.E., Kelly T., Sobolewski J. (2007). Linking Public Health, Housing, and Indoor Environmental Policy: Successes and Challenges at Local and Federal Agencies in the United States. Environ. Health Perspect..

[45] Montemurro G.R., Berry T.R., Spence J.C., Nykiforuk C., Blanchard C., Cutumisu N. (2011). “Walkable by Willpower”: Resident Perceptions of Neighbourhood Environments. Health Place.

[46] Renalds A., Smith T.H., Hale P.J. (2010). A Systematic Review of Built Environment and Health. Fam. Community Health.

[47] Gelormino E., Melis G., Marietta C., Costa G. (2015). From built environment to health inequalities: An explanatory framework based on evidence. Prev. Med. Rep..

[48] Salvo G., Lashewicz B., Doyle-Baker P., McCormack G.R. (2018). Neighbourhood Built Environment Influences on Physical Activity among Adults: A Systematized Review of Qualitative Evidence. Int. J. Environ. Res. Public Health.

[49] Wang Y., Chau C., Ng W., Leung T. (2016). A review on the effects of physical built environment attributes on enhancing walking and cycling activity levels within residential neighborhoods. Cities.

[50] Fraser S.D., Lock K. (2010). Cycling for transport and public health: A systematic review of the effect of the environment on cycling. Eur. J. Public Health.

[51] Daley M., Rissel C., Lloyd B. (2007). All dressed up and nowhere to go? A qualitative research study of the barriers and enablers to cycling in inner Sydney. Road Transp. Res..

[52] Aldred R., Watson T., Lovelace R., Woodcock J. (2019). Barriers to investing in cycling: Stakeholder views from England. Transp. Res. Part A Policy Pract..

[53] Anderson N., Bulatao R., Cohen B., National Research Council (US) Panel on Race, Ethnicity, and Health in Later Life (2004). What Makes a Place Healthy?. Neighborhood Influences on Racial/ Ethnic Disparities in Health over the Life Course., in Critical Perspectives on Racial and Ethnic Differences in Health in Late Life.

[54] McCormack G.R., Cabaj J., Orpana H., Lukic R., Blackstaffe A., Goopy S., Hagel B., Keough N., Martinson R., Chapman J. (2019). A scoping review on the relations between urban form and health: A focus on Canadian quantitative evidence. Health Promot. Chronic Dis. Prev. Can..

[55] Acevedo-Garcia D., Lochner K.A., Osypuk T.L., Subramanian S.V. (2003). Future directions in residential segregation and health research: A multilevel approach. Am. J. Public Health.

[56] Massey D.S., Rugh J.S., Steil J.P., Albright L. (2016). Riding the Stagecoach to Hell: A Qualitative Analysis of Racial Discrimination in Mortgage Lending. City Community.

[57] Kershaw K.N., Pender A.E. (2016). Racial/Ethnic Residential Segregation, Obesity, and Diabetes Mellitus. Curr. Diabetes Rep..

[58] Tamura K., Langerman S.D., Ceasar J.N., Andrews M.R., Agrawal M., Powell-Wiley T.M. (2019). Neighborhood Social Environment and Cardiovascular Disease Risk. Curr. Cardiovasc. Risk Rep..

[59] Jacobs J. (1961). The Death and Life of Great American Cities.

[60] Montgomery J. (1998). Making a city: Urbanity, vitality and urban design. J. Urban Des..

[61] Ruth M., Franklin R.S. (2014). Livability for all? Conceptual limits and practical implications. Appl. Geogr. (Sevenoaks Engl.).

