# Supplementary material for: Realtors’ Perceptions of Social and Physical Neighborhood Characteristics Associated with Active Living: A Canadian Perspective
Source: Int J Environ Res Public Health. 2020 Dec 7;17(23):9150. doi: 10.3390/ijerph17239150 (PMC7730987; doi:10.3390/ijerph17239150)
Supplement: Supplementary file 1 [file ijerph-17-09150-s001.pdf]

Realtors' Perceptions of Social and Physical Neighborhood Characteristics Associated with Active Living. A Canadian Perspective.

QUESTIONS FOR SEMI-STRUCTURED TELEPHONE INTERVIEWS

1. **Describe what you think the term "walkability" means in relation to neighbourhood design?**

    a. Where have you seen or read "walkability" or "walkable" in housing related promotional or marketing material (e.g., real estate magazines, housing sections of newspapers, television, signs and billboards)?

    b. Tell me about a time when you, or someone else in a professional setting, has mentioned the term "walkability" in conversation? Who was the conversation with and what was the nature of that conversation?

    c. If you had to describe features or attributes associated with a walkable neighbourhood to a colleague or client, what would you say?

    d. If you had to describe features or attributes associated with a low walkable neighbourhood to a colleague or client, what would you say?

    e. What might be some advantages or disadvantages of living in a walkable neighbourhood?

2. **Describe what you think the term "healthy" means in relation to neighbourhood design?**

    a. Where have you seen or read "healthy" in housing related promotional or marketing material (e.g., real estate magazines, housing sections of newspapers, television, signs and billboards)?

    b. Tell me about a time when you, or someone else in a professional setting, has mentioned the term "healthy" in conversation? Who was the conversation with and what was the nature of that conversation?

    c. If you had to describe features or attributes associated with a healthy neighbourhood to a colleague or client, what would you say?

    d. If you had to describe features or attributes associated with an unhealthy neighbourhood to a colleague or client, what would you say?

    e. What might be some advantages or disadvantages of living in a healthy neighbourhood?

3. **Describe what you think the term "cycle or bike-ability" means in relation to neighbourhood design?**

    a. Where have you seen or read "cycle or bike-ability" in housing related promotional or marketing material (e.g., real estate magazines, housing sections of newspapers, television, signs and billboards)?

    b. Tell me about a time when you, or someone else in a professional setting, has mentioned the

term “cycle or bikeability” in conversation? Who was the conversation with and what was the nature of that conversation?

c. If you had to describe features or attributes associated with a cycle-or bike-able neighbourhood to a colleague or client, what would you say?

d. If you had to describe features or attributes associated with a low cycle-or bike-able neighbourhood to a colleague or client, what would you say?

e. What might be some advantages and or disadvantages of living in a cycle-or bike-able community?

4. **Describe what you think the term “vibrancy” means in relation to neighbourhood or community design?**

   a. Where have you seen or read “vibrancy” in housing related promotional or marketing material (e.g., real estate magazines, housing sections of newspapers, television, signs and billboards)?

   b. Tell me about a time when you, or someone else in a professional setting, has mentioned the term “vibrancy” in conversation? Who was the conversation with and what was the nature of that conversation?

   c. If you had to describe features or attributes associated with a vibrant neighbourhood to a colleague or client, what would you say?

   d. If you had to describe features or attributes associated with a non-vibrant neighbourhood to a colleague or client, what would you say?

   e. What might be some advantages or disadvantages of living in a vibrant neighbourhood?

5. **Describe what you think the term “livable or livability” means in relation to neighbourhood design?**

   a. Where have you seen or read “livable or livability” in housing related promotional or marketing material (e.g., real estate magazines, housing sections of newspapers, television, signs and billboards)?

   b. Tell me about a time when you, or someone else in a professional setting, has mentioned the term “livable or livability” in conversation? Who was the conversation with and what was the nature of that conversation?

   c. If you had to describe features or attributes associated with a liveable neighbourhood to a colleague or client, what would you say?

   d. What might be some advantages or disadvantages of living in a liveable neighbourhood?
